# Phosphodiesterase type 5 inhibitors: back and forward from cardiac indications

**DOI:** 10.1007/s40618-015-0340-5

**Published:** 2015-06-28

**Authors:** C. Corinaldesi, L. Di Luigi, A. Lenzi, C. Crescioli

**Affiliations:** Department of Movement, Human and Health Sciences, Section of Health Science, Unit of Endocrinology, University of Rome “Foro Italico”, Rome, Italy; Department of Experimental Medicine, Sapienza University of Rome, Rome, Italy

**Keywords:** PDE5 inhibitors, Cardioprotection, Inflammation

## Abstract

PDE5 inhibitors (PDE5i) are widely known as treatment for erectile dysfunction (ED). This favorable action has emerged as a “side effect” from pioneering studies when PDE5i have been originally proposed as treatment for coronary artery disease (CAD). PDE5i showed marginal benefits for CAD treatment; although disappointing for that indication, they improved systemic and pulmonary vasodilation and ameliorated general endothelial function. Therefore, PDE5i have been approved and licensed also for pulmonary artery hypertension (PAH), besides ED. Nowadays, fine-tuned biomolecular mechanisms of PDE5i are well recognized to be beneficial onto myocardial contractility and geometry, to reduce tissue fibrosis, hypertrophy and apoptosis. PDE5i consistently exert benefits on heart failure, infarct, cardiomyopathy. The concept that PDE5i likely blunt Th1-driven inflammatory processes, which shift the homeostatic balance from health to disease, has emerged; PDE5i seem to decrease the release of active biomolecules from cells to tissues interested by inflammation. In this view, following clinical and basic research progresses, PDE5i can be undoubtedly “re-allocated” for cardiac indications and, hopefully, they could be approved as therapeutic tools to treat and prevent heart disease. This review aims to summarize PDE5i different clinical applications, from past to present and future, focusing on their potential power as treatment for cardiac diseases.

## Introduction

Phosphodiesterase type 5 inhibitors (PDE5i) are powerful strong vasoactive drugs widely used to treat erectile dysfunction (ED) by the specific inhibition of PDE5 activity. 3′,5′-Cyclic nucleotide phosphodiesterases (PDEs) are ubiquitous catalytic enzymes that cleave the phosphodiester bonds in cyclic adenosine or guanosine monophosphate (cAMP and cGMP) to yield 5′-cyclic nucleotides (5′AMP, 5′GMP). PDEs are a large family including at least 11 different isozymes (PDE1–PDE11), with highly conserved catalytic domain close to carboxy-terminal portion (C-terminus) [1, 2]. They share common features, such as protein sequence, structure and enzymatic properties, inhibitor sensitivity [2, 3].

The different families of mammalian PDEs are alternatively spliced in a tissue-specific manner and generate mRNAs and proteins with specific different regulatory properties. Multiple PDE isoforms are involved in creating heterogeneous cyclic nucleotide signaling within the cells [4, 5]. However, albeit some redundancy has been observed in cAMP and cGMP hydrolyzation, selective inhibitory activity has been identified on the basis of structural differences among domains: PDE5, 6 and 9 are specific for cGMP cleavage, whereas PDE4, 7 and 8 are cAMP specific; subtypes 1, 2, 3, 10 and 11 are enzymatically active on both cyclic isotypes [6, 7].

This review aims to offer an overview onto the different clinical applications of PDE5i from past, when first hypothesized for cardiovascular use, to present, mainly dedicated to ED treatment, and future, potentially “renewed” as therapeutic tools for cardiomyopathic disturbance and disease.

## PDE subfamilies, tissue distribution and function

PDE1, also known as Calcium and calmodulin dependent phosphodiesterase, is widely distributed in different tissues and cells especially in brain areas and heart [8]. PDE2, which is expressed in various tissues—i.e., adrenal cortex, brain, corpus cavernosum, heart, kidney, liver, visceral smooth muscle and skeletal muscle—hydrolyzes cGMP with higher affinity than cAMP [8, 9]. PDE3 expression is high in adipocytes, pancreatic β-cells, platelets and vascular smooth muscle. PDE3 is clinically relevant because it regulates hemodynamic parameters, i.e., increases cardiac output and reduces left and right ventricular filling pressure, through direct enhancement of myocardial contractility [10, 11]. The subfamily of PDE4 enzymes, specific for cAMP, is a therapeutic target for central nervous system since these proteins, expressed especially in the brain, are involved in several processes, such as mood control, emesis and olfactory sensory transduction [8, 9, 12]. PDE6 (photoreceptor PDE) is the best-studied enzyme due to its important role in the signal transduction of light. Like all photoreceptor, it is expressed in the outer segments of the retinal photoreceptor neurons [9]. PDE7 is widely distributed in skeletal muscle and lymphocytes and has high affinity for cAMP but still little is known about its function and regulation [8, 9]. PDE8 expression has been found particularly in testis, followed by other tissues (eye, liver, skeletal muscle, heart, 7 day embryo, kidney, ovary, and brain) [13]; it seems to be an important modulator for T effector cell functions and a regulator for T cell adhesion to vascular endothelium through the cAMP signaling pathway [14]. PDE9 is highly represented in kidney, spleen, lung, brain lymph node and thymus. It controls the activity of atrial natriuretic peptide (ANP) in excretory functions. PDE10 may be involved in the physiological regulation of motor and cognitive function [15]. Recent findings reveal that PDE11 expression is restricted in hippocampus and subiculum suggesting a potential specific role in mood and cognitive function [16].

After this summarized description of all the main types of PDEs, their localization and specific functions studied up to now, the topic of this paper will be addressed onto PDE5 function and, particularly, inhibition.

## PDE5 tissue distribution, cellular expression and subcellular localization

PDE5 subtype is generated by one specific gene, PDE5A, on chromosome 4q 25–27, with three alternative spliced variants, PDE5A1, 5A2, 5A3, which differ at 5′ ends of mRNA and amine-terminal (N-terminus) [8]. All three human PDE5 isoforms share all known functional features such as phosphorylation site, allosteric cGMP-binding sites, catalytic domain. They also have similar biochemical properties regarding, i.e., cGMP binding, cGMP hydrolysis, and drug sensitivity [17]. PDE5 is present in virtually all cell types, tissue and organs; in particular, in humans PDE5 is expressed in lung, heart, penis, vagina, uterus, brain, prostate, retina and platelets [17–29] with different and numerous functions up to the anatomical district, both in physiological and pathological conditions [17–29]. Concerning the latter ones, the inhibition of PDE5 activity can achieve different favorable effects, and, therefore, different clinical applications could be hypothesized [17–29], i.e., from urogenital diseases to neurodegenerative disorders, besides sexual dysfunction in male and females.

Table 1 summarizes tissue distribution and cell expression of PDE5 together with the possible specific clinical application of PDE5i.

**Table 1:** Tissue and cell distribution of PDE5. Potential clinical applications of PDE5i are depicted

Tissue distribution/cell expression	Potential application of PDE5i
Heart/cardiomyocytes [21]	Cardiac dysfunction (heart failure, ischemia/reperfusion injuries, infarct, ventricular arrhythmias, cardiopulmonary bypass)
Penis [17]	Erectile dysfunction
Vagina/vessel smooth muscle, endothelial and epithelial cells [18, 22]	Female sexual dysfunction
Uterus/myometrial cells [19, 22]	Preterm labor
Brain/purkinje cells [24, 25]	Psychiatric and neurodegenerative disorders
Prostate/endothelial and smooth muscle cells [26]	Lower urinary tract symptoms
Urethra/ureteral smooth muscle [22]	Ureteral colic
Bladder [22]	Overactive bladder symptoms
Lung/pulmonary arteries, vascular smooth muscle cells [23]	Pulmonary arterial hypertension
Platelets [27]	Anti-platelet agents/inhibition of platelet aggregation
Retina/endothelial and smooth muscle cells of retina and choroids vessels, ganglion and bipolar cells [28, 29]	Regulation of ocular blood flow/retinal ischemic disease

As from studies onto subcellular localization and function, PDE5 is mainly expressed and widely distributed in the cytoplasmic cell compartmentin myometrial cells, endothelial cells, smooth muscle cells of corpora cavernosa (CC) and peripheral blood mononuclear cells; in particular, in myometrial and endothelial cells, PDE5 distinct localization and accumulation in discrete vesicular compartment, corresponding to centrosomal area, may account for cGMP regulation during contraction [18–20].

## PDE5 and ED

PDE5 is widely expressed in endothelial and smooth muscle cells in penis corpora cavernosa where this specific subtype is 10–100-fold higher vs. other tissues [30] and specifically catalyzes cGMP.

### PDE5 biomolecular mechanism

Penile erection is promoted by cGMP through vascular smooth muscle cells (VSMC) relaxation mediated by the action of nitric oxide (NO).

NO, released from both nonadrenergic noncholinergic nerve terminals and endothelial cells, diffuses into VSMC and promotes cGMP synthesis [8]. Cellular cGMP level in corpus cavernosum is primarily dictated by the balance between the cGMP synthesis, by guanylyl cyclases, and cGMP shut down, by PDE5. Whenever blood flow is decreased—i.e., due to structural or functional deterioration of the peripheral vascular bed and/or nerves; NO inadequate production by neurons and/or endothelium; cGMP excessive breakdown by PDE5; VSMC decreased compliance—penile connective tissue cGMP elevation may not be adequate to provide for a sufficient degree of penile tumescence, thereby resulting in ED [31, 32]. As PDE5 specific inhibitors block the PDE5 catalytic site, cGMP stabilization and accumulation is enhanced and penile erection achieved [33, 34].

### PDE5i pharmacokinetics and efficacy

Sildenafil, vardenafil, tadalafil and avanafil, the commercialized PDE5i, are “competitive inhibitors” with cGMP for access to the catalytic site, but they do not interact with the nonhydrolytic allosteric cGMP-binding sites on PDE5 [35–37].

All PDE5i retain different bioavailability, onset of action and pharmacokinetics profiles.

Plasma half-lives of sildenafil and vardenafil are similar, about 4 h, avanafil half-life is shorter, about 3 h, whereas tadalafil half-life is longer, 17.5 h [38, 39]. Sildenafil efficacy of action can last up to 12 h, similarly to vardenafil; tadalafil efficacy is maintained for up to 36 h, while avanafil has the shorter period of efficacy, with a maximal duration of action of 6 h [40, 41].

Those PDE5i reach the highest concentrations in plasma (*T*_max_) with different times. *T*_max_ of 30–45 min for avanafil after dosing, indicating a rapid diffusion into the bloodstream when administered orally [42]; *T*_max_ values of 60 min for sildenafil, vardenafil and 120 min for tadalafil [38]. All enzymes differ for 50 % inhibitory concentration (IC_50_): 4 nM sildenafil, 0.1–0.4 nM vardenafil, 2 nM tadalafil and 4.3–5.2 nM avanafil [42]. Similar side effects are reported for all PDE5i, such as headache (10–16 %), flushing (5–12 %), dyspepsia (4–12 %), nasal congestion (1–10 %), and dizziness (2–3 %) [43].

It is generally accepted that those differences in pharmacokinetics could represent an advantage to choose and adjust the therapy on the basis of patient profile, interaction with some other drug(s) and evaluation of other aspects and components, either organic or psychic [44, 45]. However, the pharmacological properties and pharmacodynamic features per se have been considered to be criteria not enough sufficient to establish a complete evaluation on PDE5i clinical efficacy: subjective parameters, such as psychometry, patient’s preference, life quality or sexual life quality, also have to be included for a complete judgement on the effectiveness of this class of drugs [46]. Of interest, a good correlation between subjective parameters and objective biological measurements has been shown [45, 46]. On the basis of psychometric- and pharmacologic-related parameters, all three PDE5i sildenafil, tadalafil and vardenafil have been defined “harmonious” as they enable penile erection in presence of sexual desire and attractiveness, as extensively reported [46]. Correlating their relative “harmony” with pharmacokinetics helped to measure clinical therapeutic outcome: no substantial dissimilarities have been found among the three molecules. The few differences existing, i.e., between sildenafil, tadalafil and vardenafil pharmacokinetics, make tadalafil—with a longer half-life—superior in number of sexual intercourse per pill, while vardenafil and sildenafil seem preferable when duration of erection, or vascular efficacy and penile hardness are examined [46, 47].

So far, PDE5i have been demonstrated to retain a high specificity of action for ED treatment with an almost perfect tolerance profile.

## PDE5i and coronary artery disease

Albeit now widely used and specifically licensed for ED, the first clinical target of PDE5 inhibition, in pioneering researches, was angina pectoris, in view of the specific isozyme activity and expression in smooth muscle cells and platelets, where PDE5 inhibition reduces aggregation [48]. The specific PDE5i sildenafil citrate, discovered in 1989, was proposed for the treatment of coronary artery disease (CAD) [49]. Those initial studies on sildenafil demonstrated only marginal benefits due to little cardiovascular impact, although disappointing for CAD treatment, revealed interesting side effects in male subjects, as penile erection enhancement. Thereafter, further studies for this specific application led, in 1998, to the approval of sildenafil for ED treatment by the US Food and Drug Administration (FDA) [49]. As previously addressed, after sildenafil, other PDE5i, tadalafil (2003), vardenafil (2003) and, recently, avanafil (2012) have been developed and approved for ED treatment by FDA [50].

So far, for quite a long time, the use of PDE5i has been shifted away from cardiovascular applications, and it took nearly a decade after sildenafil first clinical introduction to “reallocate” this class of drugs for cardiovascular disorder treatment [48]. As previously stated, PDE5 expression is, indeed, largely diffuse in several types of tissues and cells connected with cardiovascular function, such as systemic arteries and veins, pulmonary arteries, cardiac and skeletal cells, platelets [21, 51–54].

Hence, basic and clinical studies started to focus onto vasoactive effects in tissues other than urological ones.

## PDE5i and pulmonary arterial hypertension

Due to PDE5 high expression in lung vasculature, several lines of research started to investigate the effect of PDE5 inhibition in pulmonary vascular endothelium and airway epithelium, in pulmonary arterial hypertension (PAH). PAH development and progression are strictly associated with endothelial dysfunction, vascular remodeling and fibrosis, finally leading to right heart failure. PDE5 upregulation has been documented in remodeled pulmonary artery during PAH [55] and, interestingly, PDE5 inhibition has been shown to ameliorate pulmonary artery systolic and mean artery pressure, dyspnea score and gas transfer, pulmonary vascular resistance and cardiac output, [56, 57].

It seems that circulating endothelial progenitor cell (EPC) is reduced in PAH patients [58, 59]. Remarkably, the treatment with sildenafil dose dependently rises EPC number [59]. Sildenafil-induced improvement in EPC number together with cGMP level stabilization is associated with lower mean pulmonary arterial pressure and higher cardiac index in PAH patients. Furthermore, the major vascular lesions in PAH are associated with abnormal proliferation of VSMC, involved in the pathogenesis of intimal hyperplasia. As from in vitro experiments, sildenafil prevents the proliferation and increases the apoptosis of pulmonary artery smooth cells [55]. This effect seems specific for this cellular target since in other cell types as cardiomyocytes or skeletal muscle cells PDE5 inhibition exert an anti-apoptotic effect involving Bcl/Bax signaling [60–62].

Given the improvement of several hemodynamic and clinical parameters following PDE5 inhibition, sildenafil, in 2005, and, thereafter, tadalafil have been approved by the US FDA and became first-line therapies for PAH [63], primary or secondary to other connective tissue diseases, such as scleroderma (SSc) or systemic lupus erythematosus (SLE)—which share identical pathological changes in pulmonary vessels [55].

## PDE5i and cardiomyopathies

Remarkably, in addition to the favorable actions onto PAH, PDE5 inhibition exerts cardioprotective effects as documented in different cardiac pathologies: from heart failure (HF), where sildenafil improves cardiac kinetics [64], to ischemia/reperfusion injuries, where it ameliorates vascular perfusion/density and tissue blood flow, to infarct, where it reduces damage size, apoptotic processes and cardiac hypertrophy, to ventricular arrhythmias, where it reduces disease severity, to cardiopulmonary bypass, where it increases coronary blood flow and cardiac recovery [3, 60, 61, 63].

Heart protection accomplishment by PDE5i is mainly attributed to the induced vasoactive effects linked to vascular tone regulation and release of endogenous cardioprotective molecules, such as adenosine, bradykinin and phenylephrine from endothelial cells [6, 60]. This process is related to intracellular protein kinase (PK)C/ERK activation that, in turn, triggers a signaling cascade ending in endothelial/inducible NO synthase (eNOS/iNOS) phosphorylation and NO release; following cGMP formation and stabilization, PGK activity is enhanced and results in opening K_ATP_ channel directly in mitochondria of cardiomyocytes [6]. A direct intramyocardial action of PDE5 inhibition seems to be suggested also by the results obtained in diabetic dilative cardiomyopathy after three-month treatment with 100 mg/day sildenafil: cardiac geometry and kinetics improved together with a decrease in circulating monocyte chemoattractant protein (MCP)-1 and tumor growth factor (TGF)-β, independently of any other vasodilatory or endothelial effects [65].

### PDE5i and tissue fibrosis

So far, it could be suggested that sildenafil efficacy in cardioprotection likely relies not only on vasodilation but seems, somehow, a direct effect maybe due to PDE5i-induced antifibrotic action. The latter, in fact, seems to be related to a counter-regulation of the cytokine TGF-β, which is considered an essential molecule for pathogenic stromal reorganization ending in tissue fibrosis. A deregulated TGF-β signaling is one of the driving events culminating to an altered tissue architecture reorganization [66]. Some cytokines, indeed, like MCP-1, interleukin (IL)-6 and TGF-β exert, either directly or indirectly, a decontrolled extracellular matrix (ECM) assessment and deposition, an altered fibroblast function and protein production—i.e., collagens, fibronectins or proteoglycans [67]—although the underlying mechanisms are part of a very complex network, not yet fully clarified. Furthermore, in vivo studies on fibrotic autoimmune diseases show that sildenafil exerts antifibrotic effects by inhibition of Rho kinase, a key effector for myofibroblast differentiation [68]. Those observations are in line with the ability of the PDE5i vardenafil to inhibit and reverse the *trans*-differentiation of primary human prostatic cells to myofibroblasts, that is reported to be associated with prostate stromal remodeling and lower urinary tract symptoms (LUTS) in gland hyperplasia [69, 70]. In view of those effects, the use of sildenafil is also indicated to treat digital ischemic ulcers in Raynaud’s phenomenon secondary to SSc since it decreases frequency and duration of digital vasospasms attacks—although not specifically licensed for this indication [71, 72].

Fibrosis, as well known, is a “final” process resulting from the integration of vasculopathic/immunological processes and complex cell–cell dialogue, involving, as addressed before, several active mediators and cytokines responsible for the altered ECM deposition and hypertrophy. Several lines of investigation, i.e., have documented that cardiac fibrosis development derives from a tight interaction between cardiac fibroblasts and cardiomyocytes, via direct contacts or, indirectly, via paracrine factors [73, 74]. This observation is in line with the emerging concept that cardiomyocytes and their complex communications with other nonmyocytes (from fibroblasts to endothelial or immune cells) play a pivotal role in heart pathogenesis and cardiomyopathy development [73–76].

Furthermore, based on data deriving from clinical to basic research, the central role of cardiac cells is likely particularly relevant since the early stages of cardiac diseases and not limited, i.e., to the final histopathological manifestations, such as tissue fibrosis.

The model that sees cardiomyocytes as dynamically active cells and not simply passive targets in cardiomyopathy is well in line with the emerging hypothesis that pathogenesis of heart disease (but not only) derives from the perturbation of healthy conditions by immune/inflammatory events.

### PDE5i and Th1-driven inflammation

Nowadays, inflammation has been recognized as the common link occurring at the onset of several disorders affecting different tissues, including cardiac one. Growing evidence shows that the homeostatic balance between health and disease largely depends on the immune/inflammatory status and the associated biomolecular mediators. Since quite ago, it is known that anti-inflammatory agents may have clinical benefits in preventing cardiovascular disease, in view of the direct relation existing between markers of a general inflammatory status, like C-reactive protein, and the risk of cardiovascular diseases [77].

As research progresses, new evidences emerge on the pivotal role in cardiomyopathy initiation of fine-tuned immune processes, especially driven by pro-inflammatory T helper type 1 cells (Th1) and Th1 type-related molecules and cytokines. Those mechanisms and mediators have been depicted as important triggers for disease development at very early stages, when clinical signs are still not manifested. Thus, early changes of these specific immunoactive/inflammatory molecules, detected either in peripheral blood or at tissue level, could potentially mirror a shift from healthy to disease condition at time of disease onset or even before disease initiation. In this view, we have previously reported on the critical role of the highly chemoattractant cytokine, or chemokine, interferon (IFN)γ-induced 10 kDa protein, IP-10/CXCL10 (C-X-C motif chemokine 10), in very early immune processes which elicit the inflammatory cascade, eventually culminating in cardiac homeostasis disturbance [78–80].

Chemokines are, indeed, a family of potent chemotactic small peptides known to drive and control leukocyte trafficking during inflammatory conditions underlying several allo- and autoimmune disease [80, 81]. CXCL10, as the other CXC chemokines induced by IFNγ (CXCL9/Mig, or monokine induced by interferon gamma, and CXCL11/ITAC, or interferon-inducible T cell alpha chemoattractant) is deeply involved in mechanism(s) committed to initiate and potentiate immune response, such as T cell priming [82]. Those Th1-related chemokines acting through their specific receptor CXCR3, expressed on T cell surface, retain the capacity to specifically address Th1 cell recruitment to the critical tissue sites going toward alteration induced by inflammation.

Since the pioneering studies, by Hancock et al. on heart transplantation [83], CXCL10 neutralization per se seems enough to improve cardiac function and counteract heart rejection; thereafter, the pivotal role of CXCL10 has been confirmed in human heart transplantation [78, 79, 84] and recognized in human cardiac function and diseases, from myocarditis to cardiac pulmonary bypass or coronary artery disease [85–87]. Notably, CXCL10 has been proposed as an early pretransplant predictor of organ rejection [78, 84].

In addition, human cardiomyocytes under maximal inflammatory conditions express and secrete CXCL10 besides other factors, i.e., IL-8, IL-6, virtually absent in basal normal conditions [75, 76, 86]. This observation is in line with and supports the hypothesis that cardiomyocytes act as active counterparts in inflammation-related dialogue among the different involved districts (systemic/tissues) and cells (immune/tissue resident cells). Quite remarkably, inflammatory-induced CXCL10 release by human cardiac cells is targeted by some immunesuppressors such as mycophenolate mofetil, or other drugs retaining immunomodulatory activity, such as rosiglitazone or vitamin D receptor agonists [75, 76]. From our ongoing studies, this inhibitory effect onto CXCL10 release is also observed after the treatment with sildenafil of human cardiomyocytes maximally challenged by inflammation, while no effect has been found in basal condition (personal communication, Di Luigi et al., manuscript under revision, PlosOne 2015). This in vitro observation on the anti-inflammatory effect of PDE5i is consistent with the results obtained from in vivo studies in different pathologies: PDE5i, with PDE4i and PDE7i, seem good candidates as next generation treatments in inflammatory bowel disease [88]; tadalafil and vardenafil are capable to blunt inflammation-induced IL-8 secretion by human myofibroblast prostatic cells (hBPH) [70]; PDE5i attenuate experimental autoimmune encephalomyelitis and experimental arthritis [55].

## Conclusions

Thus, besides rehabilitation of ED, there is growing interest in potential administration of PDE5i in several clinical areas, i.e., from urology, for the relief of symptoms in prostate- and bladder-related disorders, to neurology, due to protection against ischemic injury, respiratory medicine, for the effect on pulmonary hemodynamics, and rheumatology, as treatment for PAH or off-labeled treatment of Raynaud’s phenomenon in systemic scleroderma.

When evaluating PDE5i efficacy and clinical outcome in diseases other than sexual disturbance, such as cardiac diseases, to attain a reliable evaluation, several variables have to be taken in account on the basis of the evidence, as previously addressed for ED.

That is to say, while isolated cardiovascular events and sudden deaths have been reported in early studies on PDE5i, successive larger placebo-controlled clinical trials and surveillance studies documented no greater risk of this class of drugs vs. placebo: the detrimental effects were due to the incorrect PDE5i use alongside the nitro compounds (NO-donors) and, thus, contraindication with nitrate use has been assessed. Moreover, studies on effectiveness show a better cardiovascular profile of sildenafil as compared to the other PDE5i [6].

Cardiovascular disease is often concomitant with systemic inflammation and a deregulated status, as previously stated; in this light it is ingruing that sildenafil appears to be effective in un-balanced homeostasis conditions. This observation is consistent with a recent meta-analysis documenting that after sildenafil administration there are no hemodynamic effects in HF subjects with preserved ejection fraction while significant improvements have been achieved in HF patients with reduced ejection fraction [89, 90].

PDE5i seem capable to counteract the release and activity of some biomolecular mediators responsible for eliciting the shift from healthy to disease condition. Those actions are likely to mirror, at least in part, the decrease induced by cGMP stabilization of Th1-related pro-inflammatory activity [55]. Indeed, protein kinase G (PKG) activation by cGMP seems responsible for negative remodeling in HF and cardiac hypertrophy blunting. Nevertheless, PDE5 expression is very low or even undetectable in normal myocardium [90, 91] while it is markedly upregulated under inflammation-related processes, such as oxidative stress and pressure overload hypertrophy [92–96].

Overall, PDE5i efficacy in cardiovascular disturbances likely relies on different effects on heart, such as attenuation of adrenergic stimulation, reduction of ventricular-vascular stiffening and maladaptive chamber remodeling, improvement of endothelial function and enhancement of renal responsiveness [97]. So far, we could speculate that PDE5i by reverting destabilized homeostatic conditions could exert a general “anti-inflammatory” action which could be potentially associated with clinical favorable outcome.

So far, altogether, those observations might reflect and, in some measure, explain the cardioprotective effects of PDE5i, as found in animal and human studies.

Undoubtedly, other investigations in vivo and in vitro as well are mandatory to confirm the beneficial effects of PDE5i in cardiomyopathies and, possibly, clearly elucidate the underlying biomolecular mechanism(s) as potential pharmacological target(s).

Our ongoing studies are, indeed, toward this direction, and aimed to evaluate PDE5i activity in vivo on circulating level of several Th1-related chemokines, first of all CXCL10, measured in early stages of cardiomyopathies and in vitro on different human cell types, such as endothelial, cardiac and immune cells, to clarify the cellular targets. Of interest, an ongoing randomized, placebo-controlled, double-blind study (ClinicalTrial.gov, NCT01803828) enrolls diabetic females and males to evaluate PDE5i as new anti-remodeling drugs for early diagnosed and gender-dependent cardiomyopathy.

Hopefully, once well integrated researches, both at clinical and basic level, will offer strengthened and unambiguous evidence on PDE5i as anti-inflammatory safe therapeutic tools, it is conceivable that this class of drug could be licensed for the treatment or even prevention of cardiac diseases.
